# Supplementary material for: Comparative multiomics analysis of cell physiological state after culture in a basket bioreactor
Source: Sci Rep. 2022 Nov 23;12:20161. doi: 10.1038/s41598-022-24687-4 (PMC9686226; doi:10.1038/s41598-022-24687-4)

3666072.1:14389893:14390322:+@NW_023666072.1:14389893:14393114:+@NW_023666072.1:14390890:1439

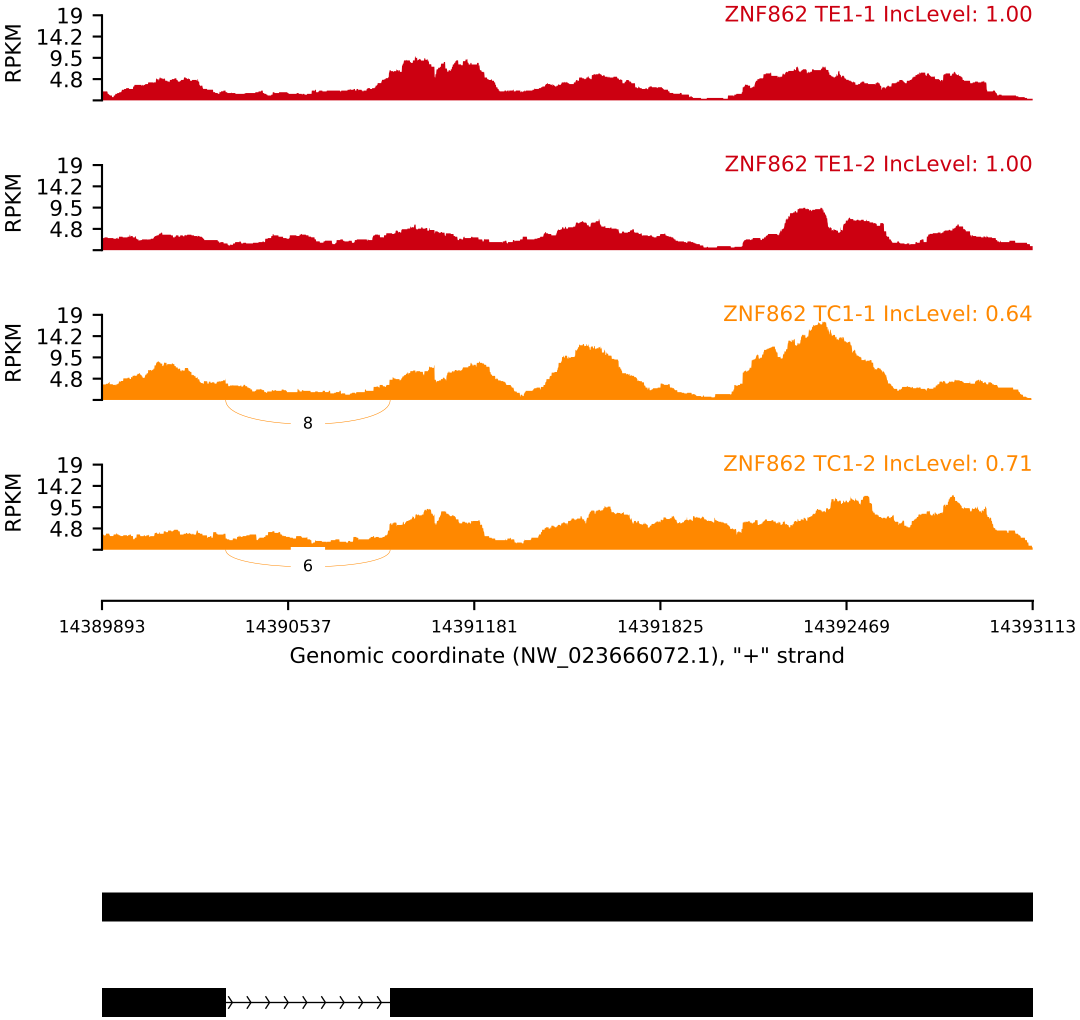

Supplement: Supplementary file 1 — Supplementary Information 1. [file 41598_2022_24687_MOESM1_ESM.zip › raw data/Transcriptomic raw data/7.AS/2.dasplot/TE1vsTC1/RI/7_ZNF862_NW_023666072.1_14389893_14390322_+@NW_023666072.1_14389893_14393114_+@NW_023666072.1_14390890_14393114_+.pdf]
